# Novel insights into weight loss: acupuncture combined with a very low-carbohydrate diet—a Swiss experience

**DOI:** 10.1177/09645284231202811

**Published:** 2023-10-03

**Authors:** Massimo Fumagalli, Raymond G Landgraaf, Nadège N Schiavi-Lods, Sorin S Golcea, Harry R Büller, Max Nieuwdorp

**Affiliations:** 1 Sinomedica, Lugano, Switzerland; 2 Department of Internal and Vascular Medicine, Academic Medical Center, Amsterdam UMC, Amsterdam, The Netherlands; 3 Medical Trials Analysis Swiss SA, Lugano, Switzerland

**Keywords:** acupuncture, complementary medicine, diabetes, diabetes and endocrinology, endocrinology, internal medicine

## Abstract

**Objective:**

The objective of this study was to assess the effects of an acupuncture–diet program for treatment of overweight and obesity.

**Methods:**

The program consisted of weekly acupuncture sessions combined with a very low-carbohydrate diet in patients with a body mass index (BMI) of 25 kg/m^2^ or above. Data were collected retrospectively between 2002 and 2021 in seven clinics in Switzerland through automated data extraction of existing medical records. The treatments described are standard care at the facilities where they took place.

**Results:**

A total of 11,233 patients were included. In those with a BMI of 25 kg/m^2^ or above, a positive effect on body weight was noted with a peak average body weight loss of approximately 17.5 kg reached after 7 months. Long-term stabilization was at about 15.5 kg after 18 months. Significant male–female differences (p < 0.01) were observed with women losing less weight. Differences were also noted between overweight, obese and extremely obese patients suggesting a BMI-dependent effect. Maximum weight loss of patients with BMI of 35 kg/m^2^ or above was 29.8 ± 12 kg, while it was 18.8 ± 8 kg for obese patients (BMI = 30–34.9 kg/m^2^) and 12 ± 7 kg for overweight patients (BMI = 25–29.9 kg/m^2^), reflecting a significant overall difference between groups (p < 0.01). Compliance to the protocol by patients and physicians seemed to be another differentiating factor; more adherent patients appeared to lose more weight and preserve body weight loss better over time.

**Conclusion:**

Although this study lacked a control group and was retrospective and observational in nature, a program of acupuncture combined with a very low-carbohydrate diet appeared to be effective at inducing weight loss among obese patients. The observed weight reduction in this retrospective chart review represents a good starting point for further investigation of this approach via a comparative evaluation.

## Introduction

The obesity pandemic is a reality as worldwide obesity has nearly tripled since 1975. In fact, in 2016, the World Health Organization (WHO) reported that more than 1.9 billion adults (above the age of 18 years) were overweight and over 650 million were obese (39% and 13% of the world’s population, respectively); mean while over 340 million children and adolescents aged 5–19 years were overweight or obese. Globally, it is considered that more than one in two adults and nearly one in six children are overweight or obese in countries from the Organisation for Economic Co-operation and Development (OECD).^1,2^ Adult obesity rates are the highest in the United States, Mexico, New Zealand and Hungary, while they are the lowest in Japan and Korea. Overweight and overeating kill more people than underweight and hunger worldwide. Globally, 8% of deaths in 2017 were the result of obesity, which represents a significant increase from 4.5% in 1990. According to the *Global Burden of Disease* study, 4.7 million people died prematurely in 2017 as a result of obesity.^2^ Overall, obesity creates a substantial economic burden in both developed and developing countries.^3^ Obesity represents a gateway to many diseases, especially type 2 diabetes mellitus, cardiovascular diseases, respiratory diseases, non-alcoholic fatty liver disease (NAFLD) and some cancers.^4^ More recently obese subjects have had an increased risk of hospitalization, serious illness and mortality due to COVID-19 infection.^5,6^

In order to lose weight, patients need to work with an interdisciplinary team of professionals to help them understand and make changes to their eating and activity habits. Treatment may consist of a mix of dietary and behavioral changes,^7^ increased physical activity, prescription of weight-loss medication and/or endoscopic procedures or even bariatric surgery. Weight-loss medication may be considered when diet and exercise programs have not worked, yet are associated with side effects including increased cardiovascular and cancer risk.^8–11^ On the contrary, endoscopic procedures and weight-loss surgery are quite effective (expected weight loss varies from 5% to 20% of total body weight for endoscopy and up to 35% or even more in case of surgery) but both are invasive, with their fair share of complications. Despite being the most effective treatment for obesity, bariatric surgery (including laparoscopic adjustable gastric band, Roux-en-Y gastric bypass, sleeve gastrectomy and biliopancreatic diversion) is marred by early and late complications, which can occur in about 17% of cases; reoperation is needed in 7% of cases.^12^

In recent years, acupuncture has emerged as a safe and potentially effective complementary treatment for obesity.^13–19^ Acupuncture involves the insertion of needles into the body, most commonly at particular traditional acupuncture point locations, followed by manual or electrical stimulation; the latter is termed electroacupuncture.^16^ Because of its clinical efficacy and cost efficiency, the use of acupuncture has been recommended by the WHO for a wide variety of diseases.^20,21^ Acupuncture is believed to induce loss of appetite, down-regulate insulin and leptin resistance and up-regulate the leptin receptor. It also lowers ghrelin levels and decreases glucose levels.^17,18,22–30^ Promising results from experimental studies and clinical evidence inspired this retrospective chart review, the aim of which was to study the effects of a method of acupuncture combined with diet in the treatment of patients with a body mass index (BMI) of 25 or above.

## Methods

### Patient population

All patients were carefully evaluated by taking a full history to understand if they would be suitable to follow this treatment protocol. Although this treatment program did not have any particular contraindications, children (below the age of 16 years), pregnant women, people with chronic renal insufficiency or heart failure and patients undergoing oncological treatment were not considered. For the purpose of this retrospective chart review, the data of existing medical records of patients with primary overweight and obesity with a BMI of 25 kg/m^2^ or above, treated by 35 physicians specializing in acupuncture across seven outpatient clinics in Switzerland during the period 2002–2021, were included and analyzed. The treatments described below are standard care at the facilities at which treatment took place. The weight-loss program consisted of weekly acupuncture sessions combined with a very low-carbohydrate diet. Patients were referred by word-of-mouth, general practitioners or medical specialists. After the first visit, all patients were offered the same outpatient treatment scheme consisting of an acupuncture–diet combination.

### Brief description of acupuncture and dietary interventions

This program combined acupuncture sessions with a very low-carbohydrate diet. Acupuncture was performed according to a specific standardized approach of weekly sessions during the weight-loss phase, once every 2 weeks during the integration phase and once a month during the maintenance phase. The style of acupuncture performed was Western medical acupuncture (WMA) and the point combinations were the same for the weight loss, integration and maintenance phases. The following traditional acupuncture point locations were chosen: CV17 (*Shanzhong*), CV12 (*Zhongwan*), CV9 (*Shuifen*) and CV4 (*Guanyuan*) on the abdomen; bilateral ST40 (*Fenglong*), SP6 (*Sanyinjiao*) and SP9 (*Yinlingquan*) on the lower legs; HT7 (*Shenmen*) on the hands; and *Sishencong* on the head. No *de qi* response was sought, and no electrical needle stimulation was performed. The acupuncture treatments lasted 30 min and incorporated a combination of frequently used point locations, with stimulation at most of them having traditionally been used to target the metabolism, immune system and neurovegetative system. The hypothesis is that acupuncture at this combination of points reduces appetite and therefore helps the patient to adhere better to the very low-carbohydrate diet (detailed below).

During the first outpatient visit, a standard medical history was collected, and weight and height registered. The physician provided a detailed explanation of the diet and handed out a list of foods that were permitted and prohibited. During the weight-loss phase, until ideal weight was reached, the diet consisted of only three meals a day (an apple or kiwi fruit for breakfast, protein (meat or fish) with vegetables or salad for lunch and some vegetables or salad for dinner). The total average daily intake was about 500 kcal. During all follow-up visits (the recommended protocol was once a week), weight was measured and registered, dietary intake was verified and acupuncture treatment was performed. This protocol was continued with the objective of reaching ideal weight (BMI within the 20–22 range). It was then followed by a phase of 2 months of stepwise integration of food items that had been prohibited in the weight-loss phase (first dairy products, then legumes and finally carbohydrates) together with acupuncture sessions once every 2 weeks. Once all food items had been reintroduced, the maintenance phase would start with a recommended acupuncture session once a month for at least 2 years and a maintenance low-carbohydrate diet with the recommendation of reducing the amount of carbohydrates to less than 20% of the total daily intake. During the integration and maintenance phases, there was no longer a strict or specific daily caloric intake, but a daily weight check was recommended during these phases as well as the acupuncture sessions. In case ideal weight was not fully achieved, the integration and maintenance phase started anyway in accordance with the patient.

### Data extraction and application of filters

Each record collected the results of a visit for a patient (ID, year of birth, sex, occupation, center, date of visit, doctor) with his or her diagnosis and a short summary—string of text—compiled by the doctor at the end of each visit.

An automated procedure was developed to extract the subject’s weight, when available, from each text string. Specifically, the algorithm was capable of detecting both the absolute weight and the relative weight variation—for example, 2 kg loss. The algorithm accounted for three different languages (Italian, French and German) and was based on searching specific keywords related to weight gain/loss in order to build the clinical history for each patient.

It was also checked whether the diagnosis text string provided information about the patients’ pathologies and height.

The analyses focused on the absolute weight loss in kg and the percentage of BMI loss with regards to the number of days since the patient’s first visit:

1. For each patient, the day of the first visit was determined with an absolute weight measurement.
2. For each follow-up visit, the days elapsed since the first visit and the weight (or % BMI) gain/loss were calculated.
3. For each discrete value (n) which defined the days elapsed since the first visit (1,2,3, . . .), we assessed all visits in the range [n − 4, n + 4].
4. The mean of the weight gain/loss (or mean % BMI gain/loss) for these visits was considered and this number (y-variable) was associated with n (x-value)—the value that was calculated as “average weight loss after 90 days” was the mean of the weight gain/loss for all the visits that were made 86, 87, 88, 89, 90, 91, 92, 93 or 94 days since the first visit. This smoothing procedure was adopted to reduce the noise of data.

The analysis was based on filters that considered only specific portions of the original data set. Four different subanalyses were performed with different filters applied.

#### Assessment of patients’ adherence

To analyze the effects of the acupuncture and diet treatment protocol according to adherence of patients with the outpatient visits, the patients were divided into two groups and data were filtered according to the number of visits performed within a specific time range. Group 1 had at least 4 visits and group 2 at least 10 visits between 2002 and 2021. This was done in order to exclude those patients who did not follow the program (drop-outs).

#### Sex differences

A specific analysis was performed based on patients’ sex.

#### Standardized protocol and differentiation in BMI subgroups

In addition, an analysis was performed in the period 2017–2021, during which the program was more protocolized and strictly followed. The standardized protocol described above was prepared and shared among all participating clinics and physicians. The treatment effect was evaluated for the different BMI categories, defined as overweight patients (BMI = 25–29.9 kg/m^2^), obese patients (BMI = 30–34.9 kg/m^2^) and extremely obese patients (BMI > 35 kg/m^2^) who had completed at least 10 visits.

#### Assessment of doctors’ adherence to the protocol

We evaluated whether the treatment depended upon the treating physician by classifying 29 physicians into three subgroups in the period 2017–2021. Six of the 35 physicians no longer practiced in the clinics during this period. The group of physicians was large and had great variation. The medical directors made a classification, based on the observations during clinical governance, dividing this heterogeneous group into three subgroups. This division was based on the number of years spent in the clinic, the number of years of acupuncture experience and the amount of internal training that had been done. In addition, the doctor’s adherence to the protocol was evaluated by the fact that some physicians did not have a sufficient proficiency with the national language and thus were potentially unable to explain the details of the diet well to the patient. Moreover, the recommended acupuncture protocol was not always strictly followed by all treating physicians. Accordingly, the three subgroups with their various characteristics were as follows:

Subgroup 1—low compliance: these physicians may have been unable to explain the diet well due to language limitations (n = 8).
Subgroup 2—medium compliance: these physicians personalized the treatment and tended not to strictly follow the treatment protocol (n = 11).
Subgroup 3—high compliance: these physicians were known to have strictly followed the treatment protocol (n = 10).

This analysis was done for patients with BMI > 35 having performed at least 10 visits over 1 year of treatment.

To assess the statistical evidence, inferential methods were applied. Student’s *t*-tests were used when comparing two (sub)groups, and analysis of variance (ANOVA) was used when comparing three subgroups. Statistical significance was defined as p < 0.05. All information collected by the physicians of the seven outpatient clinics underwent double anonymization during extraction from the medical records so that the analyzed data were not attributable to a specific person. The project does not fall within the scope of the Human Research Act (HRA) according to Article 2. Ethical approval for this retrospective chart review was obtained from the Swiss Cantonal Ethic Committee.

## Results

A total number of 11,233 patients with a BMI of 25 kg/m^2^ or higher were included and analyzed in this retrospective chart review. They were treated between 2002 and 2021 by 35 physicians across seven clinics in Switzerland. Their mean age was 49 years, mean BMI at entry was 31.2 kg/m^2^ and mean body weight was 88 kg; 27.5% were men and 72.5% were women.

### Patients’ adherence

In total, 8573 patients had at least four visits (group 1), while 4593 patients completed at least 10 visits (group 2). A total of 1933 patients had more than four but less that 10 visits and are included in group 1. Median follow-up time was 231 days for group 1 while it was 401 days for subgroup 2. For group 1, the median number of visits was 15 visits, while for group 2, the median number of visits was 23 visits.

Table 1 shows the average weight loss expressed in kg and as % of BMI reduction for the two groups over 540 days of observation.

**Table 1.** Average weight loss and % BMI reduction according to the patient’s sex and visit compliance.

Patient’s compliance	Sex	Mean age	Mean BMI	Peak average weight loss	Timepoint (days)	Average weight/% BMI loss at 90 days	Average weight/% BMI loss at 180 days	Average weight/% BMI loss at 360 days	Average weight/% BMI loss at 540 days
At least 4 visits completed (n = 8573)	Men n = 2317 (27% total)	50	32	20.5 kg	235	15 kg/13.9%	19 kg/16.8%	17 kg/15%	15 kg/13.5%
	Women n = 6256 (73% total)	49	30.8	17 kg	215	12 kg/13.8%	16.5 kg/18%	14.5 kg/16%	13 kg/14.5%
At least 10 visits completed (n = 4593)	Men n = 1194 (26% total)	51	32.88	22.5 kg	265	15.5 kg/14.5%	20 kg/18%	18 kg/16%	16 kg/14.2%
	Women n = 3399 (74% total)	49	31.29	18 kg	220	12.5 kg/14.5%	17.5 kg/19%	16 kg/18%	14 kg/15.8%

BMI: body mass index.

### Sex differences

**Figure 1.**
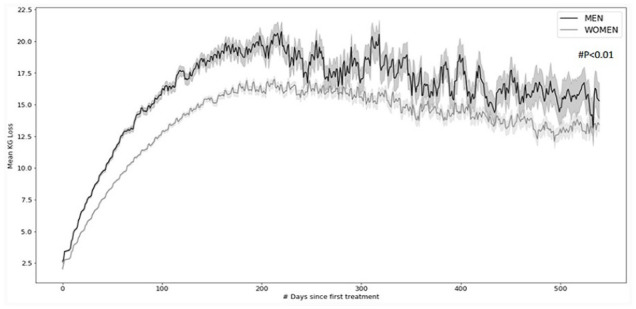
Mean weight-loss curves with standard error of mean (in gray) observed over 540 days for overweight and obese men and women treated between 2002 and 2021, having attended at least 10 visits (p < 0.01).

In both groups, women represented the majority of the patients enrolled in this weight-loss program (73% in group 1 and 74% in group 2). In both groups, women lost significantly less weight than men. In group 1, women had 17 ± 9 kg maximum weight loss (mean value ± standard deviation), whereas in men, this was 20.5 ± 14 kg (p < 0.01). In group 2, women had 18 ± 10 kg maximum weight loss, whereas men achieved a maximum weight loss of 22.5 ± 14 kg (p < 0.01; Figure 1). For group 2 (the more compliant population), the peak of average weight loss was reached after 220 days for women and after 265 days for men and was followed by a slow reduction in the average weight loss down to around 16.3 ± 14 kg for men and 14.7 ± 10 kg for women after 540 days (p < 0.01).

The average weight loss in group 1 after 90 days was 12 ± 6 kg for women and 15 ± 8 kg for men, while in group 2, it was 12.5 ± 6 kg for women and 15.5 ± 8 kg for men. For women, the peak of average weight loss (17.5 kg for all groups) was reached after 215/220 days, with slightly higher values exhibited by the groups with more visits (17 ± 10 kg for patients attending at least four visits vs 18 ± 10 kg for the group attending at least 10 visits), while for men, the peak of average weight loss (21.5 kg for all groups) was reached after 235/265 days, with higher values exhibited again by the group with more visits (20.5 ± 14 kg for patients attending at least four visits vs 22.5 ± 14 kg for the group attending at least 10 visits).

### Adherence to standardized protocol and stratification by BMI

**Figure 2.**
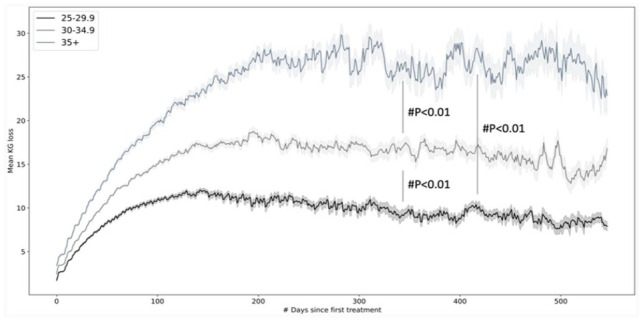
Mean weight-loss curves with standard error of mean (in gray) over 540 days for three categories of patients (with body mass index (BMI) 25.0–29.9, 30–34.9 and >35, respectively), treated between 2017 and 2021, having attended at least 10 visits (p < 0.01).

For analysis of the standardized protocol in the period 2017–2021, data from a total of 1120 overweight patients, 857 obese patients and 463 extremely obese patients were extracted from group 2 (having performed at least 10 visits). Weight-loss curves over 540 days are depicted in Figure 2. With regard to the extremely obese patients (BMI > 35), maximum weight loss was 29.8 ± 12 kg, while it was 18.8 ± 8 kg for obese patients (BMI 30–34.9) and 12 ± 7 kg for overweight patients (BMI = 25–29.9), reflecting a significant overall difference between groups (p < 0.01).

### Physician adherence

**Figure 3.**
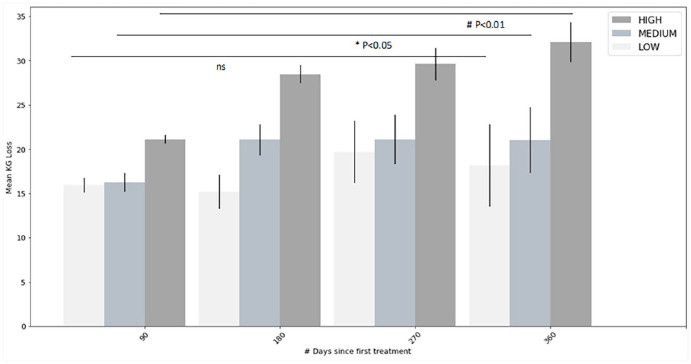
Mean weight loss over the course of 1 year (with standard error of mean) of patients with body mass index (BMI) > 35, having attended at least 10 visits, grouped according to treating physicians’ adherence to the protocol (p < 0.01 for high, p < 0.05 for medium, and p > 0.05 (non-significant) for low compliance subgroups).

Finally, the results of the analysis of compliance of the physician are represented in Figure 3. In total, data from 34 patients with low, 82 patients with medium and 288 patients with high perceived compliance of the physician were extracted. The average values recorded for each subgroup progressively increased during patients’ follow-up from 21 ± 8 kg (for the high compliance subgroup), 16 ± 7 kg (for the medium compliance subgroup) and 16 ± 4 kg (for the low compliance subgroup) at 90 days after the start of the program to 32 ± 15 kg, 21 ± 12 kg and 18.2 ± 12 kg, respectively, at 360 days after the start of the program (p < 0.01 for high, p < 0.05 for medium and p > 0.05 (non-significant) for low).

## Discussion

In line with previous studies,^17–19,30^ we observed a positive effect of the combined acupuncture–diet method on average weight loss, followed by a plateau phase of weight-loss stabilization at 540 days. Moreover, we observed that increased compliance of both the patients and physicians resulted in greater and more sustainable weight loss, especially in those with higher starting BMI and of male sex during the first 180 days of treatment. Classification into subgroups based on physician might have been arbitrary, but this division was done to analyze whether the perceived compliance of the doctor to the protocol was also a differentiating factor. Despite the high variability of results observed (e.g. due to the different physicians involved), the present data imply a beneficial effect of this treatment on weight management.

Although the positive impact on associated pathologies was not officially registered in the database, the favorable acupuncture–hypertension and acupuncture–diabetes interactions that were observed (data not shown) are well supported by other studies.^31–34^ Taken together, this supports the need for a prospective study comparing the present approach with an accepted standard treatment like an oral glucagon-like peptide (GLP)-1 analogue.

Both clinical and experimental data suggest that acupuncture exerts beneficial effects on obesity. Acupuncture seems to affect many biochemical markers of obesity such as lipid metabolism, obesity-related peptides (e.g. leptin, ghrelin) and inflammatory markers. It lowers blood glucose level by inducing insulin secretion, decreases insulin resistance and also lowers ghrelin levels and increases expression of cholecystokinin (CCK).^24,26,30,35,36^ Moreover, findings summarized in a recent paper shed light on a complex interaction of acupuncture in the regulation of appetite by the hypothalamus, although the molecular mechanism underlying these actions is only partially understood.^25^ This recent study showed that acupuncture reduces appetite due to regulation of several neurons in the arcuate nucleus of the hypothalamus (ARH), which is the main brain region for appetite regulation, and seems to regulate vagus nerve activity, modulating the vagosympathetic balance.^25^

**Figure 4.**
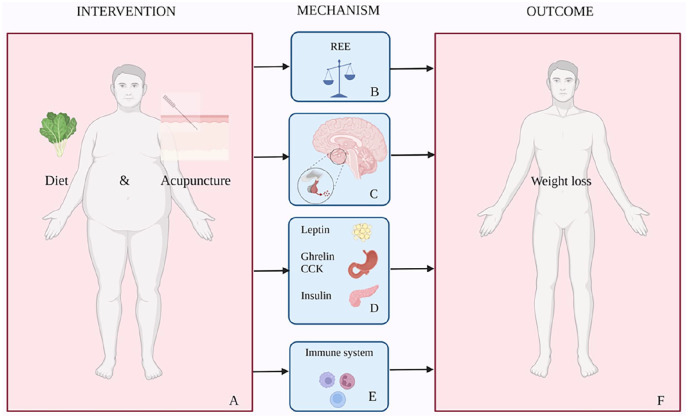
(A) Proposed mechanism of the effect of a very low-carbohydrate diet combined with acupuncture. It is a very complex process involving the metabolism, both the central and autonomic nervous systems, the gastrointestinal tract, hormones and the immune system. (B) The hypothesis is that acupuncture and diet intervention increase the resting energy expenditure (REE) or metabolic rate and induces a shift to a catabolic state. (C) Acupuncture reduces appetite due to regulation of several neurons in the arcuate nucleus of the hypothalamus, the main regulatory organ for appetite, and regulates vagus nerve activity modulating the vagosympathetic balance. (D) Acupuncture regulates lipid metabolism, down-regulates leptin resistance, lowers blood glucose level by inducing insulin secretion and decreasing insulin resistance, lowers ghrelin levels and increases the expression of cholecystokinin (CCK). (E) Acupuncture decreases inflammatory cytokines including interleukin (IL)-beta, reduces proinflammatory cytokines and reduces low grade inflammation. (F) The hypothetical result of this combined diet and acupuncture intervention is suppression of appetite, reduction of food intake, increased energy expenditure and reduction of body weight.

The proposed mechanism of the effect of this specific very low-carbohydrate diet combined with acupuncture is depicted in Figure 4. It is a complex interaction involving metabolism, both the central and autonomic nervous systems, the gastrointestinal tract, hormones and the immune system. A novel feature of this combined acupuncture–diet program is that it is hypothesized to have a synergetic effect, increasing the resting energy expenditure (REE) or metatolic rate and inducing a switch in energy balance from an anabolic to a catabolic state. This could be an additional explanation for the substantial weight loss observed in the analyzed patients. The latter, however, has not yet been supported by evidence and will be the subject of study in future research.

There are, however, several limitations to this study that need to be acknowledged. First, although our data suggest an effect on the gut-brain axis, specific data on the effect of our acupuncture method combined with this specific diet intervention on incretin and glucose metabolism are lacking. Second, most acupuncture studies in obesity have methodological limitations, including small sample size. The present analysis was a retrospective chart review of existing medical records of our current daily practice; it therefore lacks a proper control group and is not prospective. Third, it was not possible to dissect out the individual effects of diet versus acupuncture on weight.

Nevertheless, our data underscore the notion that acupuncture combined with a dietary intervention appears to have long-term beneficial effects on weight. The main strength of this retrospective chart review is the very large sample size, the long follow-up time and the standardized acupuncture approach. Further research is needed to unveil the potential relationship between acupuncture and the gut–brain axis in human obesity. Ideally, a rigorous randomized clinical trial with standardized criteria would further evaluate this minimally invasive therapy as a potential complementary or alternative treatment for obesity.

## References

[1] OECD Obesity update 2017, https://www.oecd.org/health/health-systems/Obesity-Update-2017.pdf.

[2] World Health Organization: obesity and overweight, https://www.who.int/news-room/fact-sheets/detail/obesity-and-overweight (accessed 9 June 2021).

[3] Tremmel M Gerdtham UG Nilsson PM , et al. Economic burden of obesity: a systematic literature review. Int J Environ Res Public Health 2017; 14(4): 435. 28422077 10.3390/ijerph14040435 PMC5409636

[4] Frühbeck G Toplak H Woodward E , et al. Obesity: the gateway to ill health—an EASO position statement on a rising public health, clinical and scientific challenge in Europe. Obes Facts 2013; 6(2): 117–120. 23548858 10.1159/000350627 PMC5644725

[5] Goossens GH Dicker D Farpour-Lambert NJ , et al. Obesity and COVID-19: a perspective from the European Association for the study of obesity on immunological perturbations, therapeutic challenges, and opportunities in obesity. Obes Facts 2020; 13(4): 439–452. 32791497 10.1159/000510719 PMC7490508

[6] Santosh Kumar KY Bhat PKR Sorake CJ. Double trouble: a pandemic of obesity and COVID-19. Lancet Gastroenterol Hepatol 2021; 6(8): 608. 10.1016/S2468-1253(21)00190-4 PMC8266268 34246353

[7] Ludwig DS Aronne LJ Astrup A , et al. The carbohydrate-insulin model: a physiological perspective on the obesity pandemic. Am J Clin Nutr 2021; 114: 1873–1885. 34515299 10.1093/ajcn/nqab270 PMC8634575

[8] Belviq, Belviq XR (lorcaserin) by Eisai: drug safety communication—FDA requests withdrawal of weight-loss drug, 2020, https://www.fda.gov/safety/medical-product-safety-information/belviq-belviq-xr-lorcaserin-eisai-drug-safety-communication-fda-requests-withdrawal-weight-loss-drug#:~:text=ISSUE%3A%20FDA%20has%20requested%20that,to%20voluntarily%20withdraw%20the%20drug.

[bibr9-09645284231202811] Rucker D Padwal R Li SK , et al. Long term pharmacotherapy for obesity and overweight: updated meta-analysis. BMJ 2007; 335: 1194–1199. 18006966 10.1136/bmj.39385.413113.25 PMC2128668

[bibr10-09645284231202811] Wood S. Diet drug orlistat linked to kidney, pancreas injuries. Medscape News, https://www.medscape.com/viewarticle/740855#:~:text=Among%20953%20new%20orlistat%20patients,prescription%20(p%3D0.01) (accessed 26 April 2011).

[11] Hsia DS Grove O Cefalu WT. An update on sodium-glucose co-transporter-2 inhibitors for the treatment of diabetes mellitus. Curr Opin Endocrinol Diabetes Obes 2017; 24(1): 73–79. 27898586 10.1097/MED.0000000000000311 PMC6028052

[12] Chang SH Stoll CR Song J , et al. The effectiveness and risks of bariatric surgery: an updated systematic review and meta-analysis, 2003–2012. JAMA Surg 2014; 149(3): 275–287. 24352617 10.1001/jamasurg.2013.3654 PMC3962512

[13] Lacey JM Tershakovec AM Foster GD. Acupuncture for the treatment of obesity: a review of the evidence. Int J Obes Relat Metab Disord 2003; 27(4): 419–427. 12664074 10.1038/sj.ijo.0802254

[bibr14-09645284231202811] Cho SH Lee JS Thabane L , et al. Acupuncture for obesity: a systematic review and meta-analysis. Int J Obes 2009; 33(2): 183–196. 10.1038/ijo.2008.269 19139756

[bibr15-09645284231202811] Sui Y Zhao HL Wong VC , et al. A systematic review on use of Chinese medicine and acupuncture for treatment of obesity. Obes Rev 2012; 13(5): 409–430. 22292480 10.1111/j.1467-789X.2011.00979.x

[bibr16-09645284231202811] Abdi H Zhao B Darbandi M , et al. The effects of body acupuncture on obesity: anthropometric parameters, lipid profile, and inflammatory and immunologic markers. Scientific World J 2012; 2012: 603539. 10.1100/2012/603539 PMC3353309 22649299

[bibr17-09645284231202811] Fang S Wang M Zheng Y , et al. Acupuncture and lifestyle modification treatment for obesity: a meta-analysis. Am J Chin Med 2017; 45(2): 239–254. 28231746 10.1142/S0192415X1750015X

[bibr18-09645284231202811] Belivani M Lundeberg T Cummings M , et al. Immediate effect of three different electroacupuncture protocols on fasting blood glucose in obese patients: a pilot study. Acupunct Med 2015; 33(2): 110–114. 25522743 10.1136/acupmed-2014-010662

[19] Chen J Chen D Ren Q , et al. Acupuncture and related techniques for obesity and cardiovascular risk factors: a systematic review and meta-regression analysis. Acupunct Med 2020; 38(4): 227–234. 32310001 10.1136/acupmed-2018-011646

[20] World Health Organization. Acupuncture: review and analysis of reports on controlled clinical trials. Geneva: World Health Organization, 2003.

[21] World Health Organization. WHO global report on traditional and complementary medicine, 2019, https://apps.who.int/iris/handle/10665/312342

[22] Gong M Wang X Mao Z , et al. Effect of electroacupuncture on leptin resistance in rats with diet-induced obesity. Am J Chin Med 2012; 40(3): 511–520. 22745067 10.1142/S0192415X12500395

[bibr23-09645284231202811] Ito H Yamada O Kira Y , et al. The effects of auricular acupuncture on weight reduction and feeding-related cytokines: a pilot study. BMJ Open Gastroenterol 2015; 2(1): e000013. 10.1136/bmjgast-2014-000013 PMC4599151 26462269

[bibr24-09645284231202811] Wang LH Huang W Wei D , et al. Mechanisms of acupuncture therapy for simple obesity: an evidence-based review of clinical and animal studies on simple obesity. Evid Based Complement Alternat Med 2019; 2019: 5796381. 30854010 10.1155/2019/5796381 PMC6378065

[bibr25-09645284231202811] Wang L Yu CC Li J , et al. Mechanism of action of acupuncture in obesity: a perspective from the hypothalamus. Front Endocrinol 2021; 12: 632324. 10.3389/fendo.2021.632324 PMC8050351 33868169

[bibr26-09645284231202811] Zhao ZM Liu CL Zhang QY , et al. Acupuncture treatment reduces body weight possibly by down-regulating insulin and leptin resistance, and up-regulating soluble leptin receptor level in prediabetic patients. Zhen Ci Yan Jiu 2018; 43: 506–511. 30232854 10.13702/j.1000-0607.180184

[bibr27-09645284231202811] Chen C Wang HC Zhai X , et al. Progress of research on mechanism of acupuncture for diabetes mellitus. Zhen Ci Yan Jiu 2018; 43(9): 601–605. 30232872 10.13702/j.1000-0607.180009

[bibr28-09645284231202811] Li ZX Zhang HH Lan DC , et al. Progress of researches on mechanisms of acupuncture therapy for insulin resistance. Zhen Ci Yan Jiu 2019; 44(3): 231–234. 30945509 10.13702/j.1000-0607.180057

[bibr29-09645284231202811] Park KS Park KI Suh HS , et al. The efficacy and safety of acupuncture on serum leptin levels in obese patients: a systematic review and meta-analysis. Europ J Integr Med 2017; 11: 45–52.

[30] Belivani M Dimitroula C Katsiki N , et al. Acupuncture in the treatment of obesity: a narrative review of the literature. Acupunct Med 2013; 31(1): 88–97. 23153472 10.1136/acupmed-2012-010247

[31] Zhong YM Luo XC Chen Y , et al. Acupuncture versus sham acupuncture for simple obesity: a systematic review and meta-analysis. Postgrad Med J 2020; 96(1134): 221–227. 32015189 10.1136/postgradmedj-2019-137221 PMC7146934

[bibr32-09645284231202811] Firouzjaei A Li GC Wang N , et al. Comparative evaluation of the therapeutic effect of metformin monotherapy with metformin and acupuncture combined therapy on weight loss and insulin sensitivity in diabetic patients. Nutr Diabetes 2016; 6(5): e209. 10.1038/nutd.2016.16 PMC4895377 27136447

[bibr33-09645284231202811] Chen C Liu J Sun M , et al. Acupuncture for type 2 diabetes mellitus: a systematic review and meta-analysis of randomized controlled trials. Complement Ther Clin Pract 2019; 36: 100–112. 31383426 10.1016/j.ctcp.2019.04.004

[34] Yang M Yu Z Chen X , et al. Active acupoints differ from inactive acupoints in modulating key plasmatic metabolites of hypertension: a targeted metabolomics study. Nature 2018; 8: 17824. 10.1038/s41598-018-36199-1 PMC6292875 30546033

[35] Güçel F Bahar B Demirtas C , et al. Influence of acupuncture on leptin, ghrelin, insulin and cholecystokinin in obese women: a randomised, sham-controlled preliminary trial. Acupunct Med 2012; 30(3): 203–207. 22729015 10.1136/acupmed-2012-010127

[36] Zhu FY Li ZM Tang LJ , et al. Progress of the mechanism research of acupuncture in treatment of diet-induced obesity. Zhen Ci Yan Jiu 2020; 45(3): 255–259. 32202720 10.13702/j.1000-0607.180489

